# The Association of Menopausal Symptoms and Social Support Among Saudi Women at Primary Health Care Centers in Taif, Saudi Arabia

**DOI:** 10.7759/cureus.26122

**Published:** 2022-06-20

**Authors:** Bashaer Aloufi, Noha S Hassanien

**Affiliations:** 1 Preventive Medicine, Saudi Board of Preventive Medicine, Taif, SAU; 2 Biostatistics, Alexandria University, Alexandria, EGY

**Keywords:** lifestyle factors, menopause symptoms, saudi women, social support, menopause rating scale

## Abstract

Background

Menopause is a challenging period for all women. The severity of menopausal symptoms hurts their quality of life and daily activity. The aim of this study was to investigate whether the severity of menopausal symptoms are associated with social support and lifestyle factors among Saudi women to help policymakers develop the appropriate intervention health program.

Methods

A cross-sectional study of 361 middle-aged Saudi women was conducted through interviews using a valid and reliable questionnaire. The menopause rating scale (MRS) was used to determine the severity of menopausal symptoms and the multidimensional scale of perceived social support (MSPSS) was used to assess perceived social support among females. Linear regression was conducted to assess the association between MRS scores and MSPSS scores after adjustment of covariates.

Results

The mean total menopause rating scale was 13.7 *± *8.3. Physical and mental exhaustion (80.3%), joint and muscular discomfort (79.2%), and irritability (75.9%) were the most prevalent menopausal symptoms for all women. The mean MSPSS was calculated as 4.3 *±*1.8. Perceived social support and lifestyle factors were significant predictors of menopausal symptoms.

Conclusions

Postmenopausal Saudi women complain of a variety of physical and psychological symptoms. The current study shows that social support, quitting smoking, losing weight, and increasing physical exercise can help to alleviate or reduce many of the unpleasant symptoms of menopause. This evidence will help policymakers design health intervention for this age group.

## Introduction

Menopause is defined as the cessation of monthly cycles for 12 months that occurs naturally in the majority of women and is linked to the gradual decrease of ovarian follicles and hormonal changes. It is a natural transition that occurs in all females after their mid-forties [1]. The female's view of menopause should not be underestimated, it marks the end of reproductive ability and the start of the aging process. Due to the decrease in estrogen hormone, women feel compromised physical well-being along with several menopausal symptoms such as psychological, physical, sexual, and vasomotor complaints during menopause [2].

The symptoms of menopause are quite varied, and different countries have reported a wide range of symptoms with variable degrees of severity depending on race and ethnicity [3,4]. The most prevalent symptoms include joint and muscle pain, hot flashes, nervousness, depression, insomnia, and general fatigue [3-5]. El Sherbini et al. established that there are urogenital symptoms that may entail sexual problems, dryness of the vagina, and bladder problems that may occur due to aging [6]. As per 2017 statistics, the Saudi Arabian population (20,408,362) indicates that nearly half the population is female (49.06%) [7]. A majority of Saudi women reach menopause between 51 and 55 years of age [8]. Also, reproductive health is one of the hot topics on the agenda of Saudi Vision 2030 [9]. Statistically, Saudi females older than 65 years represent 51.1% of the population [7]. Several tools to assess the severity of menopausal symptoms are available. One of the most commonly used in literature is the menopausal rating scale (MRS) and it is considered the standard tool because of its good psychometric property and ease of applicability [10].

The literature documented several determinants of menopausal symptoms such as socio-demographic variables, and psychosocial, cultural, social, and lifestyle factors. Those factors influence the prevalence and severity of menopausal symptoms. One of the factors that have recently been a matter of concern is the perceived social support among females. Research highlights that women need social support to cope and adapt to the menopausal symptoms in this stressful period of their life, and this may help control their symptoms [11,12]. The severity of symptoms may have a negative impact on daily activity and quality of life. Based on life expectancy, women spend about one-quarter of their life in the menopausal period, so it is crucial to assess the prevalence, severity, and determinants of those symptoms to establish required targeted health interventions and specific geriatric health services to deal with the menopausal health problems [13].

Studies to assess the effect of perceived social support and lifestyle factors such as obesity, physical activity, and smoking on the severity of menopausal symptoms among Saudi Arabian women are lacking. In addition, the results of studies regarding those correlates of severity of menopausal symptoms showed diversity all over the world. Consequently, we performed this study to determine the collective influence of those modifiable factors on the severity of menopausal symptoms among Saudi women in the middle-age group to analyze the potential effect of perceived social support to help policymakers develop suitable health intervention programs to alleviate the severity of symptoms and enhance the quality of life of menopausal women.

## Materials and methods

Study participant and sampling technique 

An analytical cross-sectional study was carried out among females aged 45 to 65 years attending the primary health care (PHC) centers of Taif, Saudi Arabia. These PHC centers were chosen as they are the first line of service as per the Saudi health care system transformation plan, and serve a huge number of women coming in for gynecological and non-gynecological check-ups. The required sample size was calculated to be 361 females, using a correlation coefficient of 0.14 between the total menopausal rating score and total perceived social support scale from a previous study [14], with 5% precision using and 80% power using G*Power software version 3.1.9.4. Taif is divided into four geographic areas, and one primary health care center was chosen randomly from each of these four regions based on a list of centers obtained from the Taif health directorate. Then from each center, we randomly selected 90 women using a systematic random sample of every third female till fulfilling the required sample size. We excluded females who were pregnant, lactating, on medications such as anxiolytics, antidepressants, antipsychotic drugs, or those who had hysterectomy or oophorectomy or had been diagnosed with any type of cancer.

Data collection

Data was collected through face-to-face interviews using a structured anonymous pre-tested questionnaire for females who met inclusion criteria and agreed to participate during the period from January to April 2022. The questionnaire was tested in a pilot study of 30 women. The questionnaire comprised four sections (see Appendices).

Section one surveyed socio-demographic data (age, marital status, education, and occupation), reproductive history (parity, regularity of menstrual cycle), lifestyle factors (smoking of cigarette or shisha, physical activity), and chronic disease, and anthropometric measurements such as height and weight were measured. Body mass index (BMI) was calculated and classified based on WHO criteria (underweight, BMI< 18.5 kg/m2; normal weight, 18.5 ≤ BMI ≤ 23.9 kg/ m2; overweight, 24 ≤ BMI ≤ 27.9 kg/ m2; obesity, BMI ≥ 28.0 kg/m2).

In section two, the Arabic version of the menopause rating scale (MRS) which was validated in a previous study was used to assess the prevalence and severity of menopausal symptoms [15,16]. Mean scores of menopausal categories were compared for different symptoms. It is composed of 11 symptoms in three subscales, namely the somatic subscale (hot flushes, sleep problems, heart discomfort, joint, and muscle pain); psychological subscale (anxiety, depressed mood, irritability, physical and mental exhaustion); urogenital subscale (sexual problems, vaginal dryness, and bladder problems). Each symptom was scored from none (0) to very severe (4). The total MRS score was calculated by summation of all subscales yielding a score range from 0 to 44. Then, the severity of symptoms was classified as none/little (0-4), mild (5-8), moderate (9-16), and severe/very severe (17 or more). The scale had good reliability (Cronbach’s alpha = 0.880) in our target population.

The third section assessed physical activity (PA) using the Arabic version of the International Physical Activity Questionnaires (IPAQ) [17]. The total score was obtained by summation of the duration in minutes and frequency in days of walking, moderate-intensity, and vigorous-intensity activities. Physical activity was categorized as low, moderate, and high.

Section four focused on social support using the valid multidimensional scale of perceived social support scale (MSPSS) [18]. It consists of three domains of support (family, friends, and a significant other). Each subscale included four items. We calculated the mean subscale scores which were categorized as low support (1 to 2.9), moderate support (3 to 5), and high support (5.1 to 7). By adding all items the total perceived social support score was obtained. The scale had excellent reliability (Cronbach’s alpha = 0.962) in our target population.

Ethical issues

The authors obtained institutional ethical approval from King Abdelaziz City for Science and Technology (IRB approval number: 314, Registration number: HAP-02-T-067). A participant's consent was required before participating and confidentiality was ensured. The study was conducted according to the Declaration of Helsinki.

Statistical analysis

The Statistical Package for Social Sciences (SPSS) version 23.0 (IBM Corp., Armonk, NY, USA) software was used for analysis. We calculated frequencies and percentages for categorical variables. For quantitative variables, descriptive statistics were calculated as means and standard deviations (SDs) or medians and interquartile ranges (IQRs). The Shapiro-Wilk test was used to determine the normality of the data. The following tests were used for non-parametric analysis: Mann- Whitney test (two or more groups different), Kruskal-Wallis test (three or more groups different), and a post hoc analysis to see if there is a significant difference. We measured the correlation between two quantitative variables using the Spearman correlation coefficient. We considered a P-value of 0.05 significant and all tests had two-tailed hypotheses. Multiple linear regression analysis was conducted on all significant factors associated with the total MRS score and its subscales.

## Results

Table 1 presents the characteristics of the 361 female respondents to the questionnaire. The mean age was 53.6±5.8 years. About 36.3 % of the women were overweight and 43.5 % were obese. The majority of them were married and had a university educational level (75.1% and 70.9%, respectively). About 71% of women were not working. Most of them (90.9%) were parous. About 88% of participants were non-smokers and only 8% were current smokers. More than half of them (59.8%) had no chronic diseases. Among those who had chronic diseases, the most frequent diseases were hypertension (21.9%), diabetes (13.6%), hyperlipidemia (6.1%), bronchial asthma (4.2%), and osteoporosis (3.3%). Almost three fourth of them (72.9%) had irregular menses in the past twelve months. About 43.2% of females had low physical activity, whereas 38%, and 18%, engage in moderate and high levels of physical activity, respectively.

**Table 1:** Characteristics of participants

Characteristics	Frequency N=361	Percentage
Age in years		
45-	99	27.4
50-	106	29.4
55-	82	22.7
60-65	74	20.5
Mean ± SD	53.6±5.8	
BMI		
Normal	73	20.2
Over-weight	131	36.3
Obese	157	43.5
Mean ± SD	30.1±6.7	
Marital status		
Single	19	5.3
Married	271	75.1
Divorced	36	10.0
Widowed	35	9.7
Educational level		
Uneducated/primary	13	3.6
intermediate	24	6.6
Secondary	68	18.8
University	256	70.9
Occupation		
Not working	255	70.6
Working	106	29.4
Fertility		
Nulliparous	33	9.1
Parous	328	90.9
Smoking		
Non-smoker	317	87.8
Ex-smoker	15	4.2
Current smoker	29	8.0
Chronic diseases		
No	216	59.8
Yes	145	40.2
· Diabetes	49	13.6
· Hypertension	79	21.9
· Heart disease	5	1.4
· Hyperlipidemia	22	6.1
· Asthma or allergy	15	4.2
· Osteoporosis	12	3.3
Regularity of menstrual cycle in the past 12 months		
Regular	98	27.1
Irregular	263	72.9
Physical activity		
Low activity	156	43.2
Moderate	137	38.0
High	68	18.8

Table 2 shows the frequency of menopausal symptoms as assessed by the MRS. The most prevalent menopausal symptoms for all women (n=361) were physical and mental exhaustion (80.3%), with 35.2 % of them having mild symptoms. The second most frequent symptom was joint and muscular discomfort (79.2%) with most of them having mild to moderate symptoms (29.6%). The third common symptom was irritability (75.9%). Depressive mood and hot flushes ranked as the fourth most common symptom (68.6%) while sleep problems came in fifth (68.4%) and sexual problems came in sixth (65.9%). As per MRS, 40.2% of females had severe symptoms, 31.3% had moderate symptoms and 14.1% had mild symptoms. The mean total MRS was 13.7±8.3.

**Table 2:** Descriptive statistics of menopausal symptoms among participants Sd: Standard deviation, IQR: Interquartile range, MRS: Menopause rating scale

Item	None No. (%)	Mild No. (%)	Moderate No. (%)	Severe No. (%)	very severe No. (%)	Total No. (%)
Somatic MRS Mean ±Sd, median (IQR)	5.1 ±3.2, 5 (3-7)					
· Hot flushes, sweating	113	83	104	46	15	248
	(31.3)	(23.0)	(28.8)	(12.7)	(4.2)	(68.6)
· Heart discomfort	831	103	59	15	1	178
	(50.7)	(28.5)	(16.3)	(4.2)	(.3)	(49.3)
Trouble sleeping	114	87	106	41	13	247
	(31.6)	(24.1)	(29.4)	(11.4)	(3.6)	(68.4)
Joint and muscular discomfort	75	100	108	50	28	286
	(20.8)	(27.7)	(29.9)	(13.9)	(7.8)	(79.2)
Psychological MRS Mean ±Sd, median (IQR)	5.4 ±3.7, 5 (2-8)					
· Depressive mood	113	103	88	45	12	248
	(31.3)	(28.5)	(24.4)	(12.5)	(3.3)	(68.6)
· Irritability	87	109	101	47	17	274
	(24.1)	(30.2)	(28.0)	(13.0)	(4.7)	(75.9)
· Anxiety	124	108	80	37	12	237
	(34.3)	(29.9)	(22.2)	(10.2)	(3.3)	(65.6)
· Physical and mental Exhaustion	71	127	107	41	15	290
	(19.7)	(35.2)	(29.6)	(11.4)	(4.2)	(80.3)
Urogenital MRS Mean ±Sd, median (IQR)	3.3 ±2.7, 3 (1-5)					
· Sexual problems	123	90	92	40	16	238
	(34.1)	(24.9)	(25.5)	(11.1)	(4.4)	(65.9)
· Bladder problems	155	100	63	32	11	206
	(42.9)	(27.7)	(17.5)	(8.9)	(3.0)	(57.0)
· Dryness of vagina	157	90	65	40	9	204
	(43.5)	(24.9)	(18.0)	(11.1)	(2.5)	(56.5)
Total MRS categories Mean ±Sd, median (IQR)	52	51	113	145	-	-
	(14.4)	(14.1)	(31.3)	(40.2)	-	-
	13.7±8.3, 13 (7-19)					

Table 3 shows the distribution of the MSPSS. Overall, 42.5% of participants had moderate social support and 34.8 % had high support. The mean of the total social support scale was 4.3± 1.8 which was considered a moderate level of social support. Concerning the subscales of MSPSS, the highest percentage of females had moderate social support regarding the significant other and friend subscale (49.6% and 38%, respectively). While for the family subscale the highest percentage of them (46.8%) had high social support. 

**Table 3:** Descriptive statistics of the multidimensional scale of perceived social support (MSPSS) Sd: Standard deviation, IQR: Interquartile range

	Frequency	(%)
Significant Other subscale		
· Low support	80	(22.2)
· Moderate support	179	(49.6)
· High support	102	(28.3)
Family subscale		
· Low support	74	(20.5)
· Moderate support	118	(32.7)
· High support	169	(46.8)
Friends subscale		
· Low support	105	(29.1)
· Moderate support	137	(38.0)
· High support	119	(33.0)
Total MSPSS		
· Low support	80	(22.7)
· Moderate support	150	(42.5)
· High support	123	(34.8)
Mean ±Sd, median (IQR)	4.3± 1.8 , 4.5 (2.8-6.0)	

Table 4 shows the factors affecting MRS and its subscales. The average MRS was significantly lower in women aged between 45 to 50 than in other age groups (p=0.000). Both overweight and obese women were significantly higher in average MRS than normal-weight women (p=0.024). Non-working women had significantly higher average MRS (p=0.017). Current smokers and ex-smokers had significantly higher average MRS than non-smokers (p=0.011). Those with non-regular menses had significantly higher average MRS than those with regular menses (p=0.000). Women with low physical activity had a significantly higher average menopause rating than those with moderate and high physical activity (p=0.025). Regarding the somatic subscale, old age, high BMI, non-working, smoking, and irregular menses were significantly associated with high somatic symptom scores. However, only smoking and low physical activity were significantly associated with a higher psychological subscale (p=0.039 and 0.009, respectively). As per the urogenital subscale, older age, high BMI, marital status, non-working, parous, smoking, irregular menses, and low physical activity were significantly associated with higher scores.

**Table 4:** Factors associated with the MRS and its subscales *Significant at p ≤ 0.05 BMI: Body mass index, SD: Standard deviation, IQR: Interquartile range, MRS: Menopause rating scale

Factors	Somatic				Psychological					Urogenital			Total		
	Mean ± SD	Median (IQR)	P-value	Mean ± SD		Median (IQR)		P-value	Mean ± SD		Median (IQR)	P-value	Mean ± SD	Median (IQR)	P-value
Age															
45-	3.4±0.019	3 (1-6)	0.000*	5.1 ±.04		5 (1-8)		0.228	2.5± .47		2 (0-4)	0.000*	10±7.9	11(3-17)	0.000*
50-	5.2± 0.95	5 (3-7)		5.3± .29		5 (3-7)			3.1 ±.57		3 (1-5)		13±7.3	13 (9-18)	
55-	5.5± 3.3	5 (3-8)		5.3 ±.50		4 (2-8)			3.9 ±.67		3.5 (2-6)		14.9± 1.3	14 (6-21)	
60-65	6.3 ± 3.1	6 (4-9)		5.8± 3.2		6 (3-8)			4.1±.0.01		4 (2-6)		16.1 ±7.1	16 (11-21)	
BMI															
Normal	4.3 (3)	4 (6-2)	0.036*	4.6 (3.3)		4 (7-2)		0.108	2.7 (2.7)		2 (4-0)	0.003*	11.6 (6.8)	12 (17-6)	0.024*
Overweight	4.8 (3.4)	4 (7-2)		5.2 (3.6)		5 (8-2)			3.1 (2.5)		3 (5-1)		13 (8.1)	12 (19-7)	
Obese	5.5 (3.2)	5 (8-3)		5.9 (4)		5 (8-3)			3.8 (2.8)		3 (6-1)		15.2 (8.8)	14 (21-8)	
Marital															
Single	4.4 (3.4)	3 (8-2)	0.791	5.7 (4.3)		5 (9-3)		0.842	2 (2.1)		2 (3-0.0)	0.006*	12.1( 8.2)	10 (17-6)	0.549
Married	5 (3.2)	5 (7-3)		5.3 (3.7)		5 (8-3)			3.5 (2.7)		3 (6-1)		13.9 (8.2)	13 (20-8)	
Divorced	5.2 (3.4)	4 (8-3)		4.8 (3.4)		4 (6-4)			2.2 (2.4)		2 (3-0)		12.3 (7.3)	12 (3-0)	
Widowed	5.2 (3.6)	5 (8-2)		5.3 (4.3)		5 (8-1)			3.6 (2.9)		4 (5-1)		14.1 (9.5)	15 (19-7)	
Educational Level															
Uneducated/Primary	5.7 (4.4)	7 (8-1)	0.245	4.9 (5)		4 (7-0.5)		0.853	4.2 (3.7)		4 (7-1)	0.793	14.8 (12.3)	17 (22-3)	0.761
Intermediate	4.4 (3.5)	4.5 (7-1)		5.1 (3.8)		5.5 (8-1)			3.6 (3)		3 (5-1)		13.1 (9.9)	14.5 (20-3)	
Secondary	5.7 (3.3)	5 (8-3)		5.4 3.7)		5 (8-2)			3.5 (2.9)		3 (5-1)		14.5 (8.3)	14 (20-9)	
University	4.9 (3.2)	4 (7-3)		5.4 (3.7)		5 (8-3)			3.2 (2.6)		3 (5-1)		13.5 (7.9)	12 (19-7)	
Occupation															
Not working	5.3 (3.3)	5 (8-3)	0.002*	5.4 (3.8)		5 (8-2)		0.954	3.7 (2.8)		3 (6-1)	0.000*	14.4 (8.4)	14 (20-8)	0.017*
Working	4.2 (3.1)	4 (6-2)		5.3 (3.7)		5 (8-2.7)			2.5 (2.4)		2 (4-0)		12.1 (7.7)	11 (17-7)	
Fertility															
Nulliparous	5.1 (3.2)	5 (7-3)	0.179	5.3 (3.7)		5 (8-2)		0.777	3.4 (2.7)		3 (5-1)	8.046*	13.8 (8.2)	13 (19-8)	0.328
Parous	4.4 (3.5)	4 (7.5-1.5)		5.6 (4.1)		5 (9-2)			2.5 (2.5)		2 (3-0)		12.5 (8.6)	12 (18-5)	
Smoking			0.034*					0.039*				0.009*			0.011*
Non-smoker	4.9 (3.3)	5 (7-2)		5.2 (3.7)		5 (7-2)			3.2 (2.7)		3 (5-1)		13.2 (8.0)	12 (19-7)	
Ex-smoker	4.8 (3.3)	4 (7-3)		5.9 (4.6)		6 (9-2)			3.4 (2.4)		3 (5-1)		14.1 (8.8)	14 (19-8)	
Current smoker	6.6 (3.1)	6 (8-4)		7.3 (4.3)		7 (10-3.5)			5 (3.1)		4 (7-2)		18.8 (9.0)	17 (21-14)	
Chronic disease			0.170					0.813				0.196			0.345
No	4.8 (3.3)	4 (7-2)		5.3 (3.8)		5 (8-2)			3.2 (2.7)		3 5-1)		13.3 (8.2)	13 (18-7)	
Yes	5.3 (3.3)	5 (7-3)		5.4 (3.7)		5 (8-3)			3.6 (2.8)		3 (5-1)		14.3 (8.3)	13 (20-8)	
Menses			0.000*					0.589				0.000*			0.000*
Regular	3.6 (3.1)	3 (5-1)		5.3 (4.2)			5 (8-2)		2.5 (2.7)		2 (3-0)		11.4 (8.8)	10 (16-4)	
Irregular	5.5 (3.1)	5 (8-3)		5.4 (3.6)			5 (8-3)		3.6 (2.7)		3 (6-1)		14.5 (7.9)	14 (20-8)	
Physical activity			0.200					0.009*				0.035*			0.025*
Low	5.4 (3.4)	5 (8-3)		6.1 (4)			6 (9-3)		3.7( 2.8)		3 (6-2)		15 (8.6)	14.5 (21-8)	
Moderate intensity	4.7 (3.1)	4 (7-3)		4.9 (3.5)			5 (7-2)		3.1 (2.6)		3 (5-1)		12.7 (7.8)	13 (18-6)	
High intensity	4.6 (3.2)	4 (7-2)		4.6 (3.5)			4 (7-2)		2.8 (2.7)		2 (4-0)		12 (7.7)	12 (18-6)	

Table 5 shows the results of Spearman correlation (r_s_) of total menopausal rating scales and their subscales with the total scores of the perceived social support scale and its subscales. The total MRS had a significant negative weak correlation with total social support scores. Both psychological and urogenital subscales of MRS had a significant weak correlation with the total social support scale (r_s_=-.199 p=0.000 and r_s_ =-.219 p=0.000, respectively). 

**Table 5:** Correlation between total menopausal rating scores and its subscales with perceived social support scores r_s_ (Spearman correlation coefficient)-, * significant (p-value ≤ 0.05)

	Total MRS scale	Somatic subscale	Psychological subscale	Urogenital subscale
Total social	r_s_ = -.174	r_s_ =-.040	r_s_ =-.199	r_s_ =-.219
	p=0.001*	p=0.447	p=0.000*	p=0.000*
Significant Other subscale	r_s_ =-.042	r_s_ = -.003	r_s_ =-.067	r_s_ =-.199
	p=0.429	p=0.961	p=0.205	p=0.000
Family subscale	r_s_ =-.109	r_s_ = .058	r_s_ = -.126	r_s_ =-.126
	p=0.039*	p=0.276	p=0. 016*	p=0.016*
Friends subscale	r_s_ =-.124	r_s _ =-.045	r_s_ =-.099	r_s_ =-.194
	p=0.019*	p=0.394	p=0.059	p=0.000*

The results of multiple linear regression are shown in Table 6 for significant predictors of the menopausal rating scale and its subscales. According to model 1, smoking is the most important predictor of an increase in the severity of menopausal symptoms (b=3.943, p 0.008). An increase in BMI led to an increase in MRS (b=.352, 0.000). Physical activity decrease the MRS (b=-1.065, p=0.048) and high social support led to decline in MRS (b= -.600, p=0.007). The significant predictors of increase in somatic subscale were irregularity of menstrual cycle in the past 12 months, smoking, high BMI, and older age of females, and in that magnitude of order. Concerning the psychological subscale, smoking increases the severity of symptoms (b=.839, p=0.013). However, both physical activity and high social support led to its decline (b=-.637, p=0.013 and b =-.419, p=0.000, respectively). As per the urogenital subscale, both smoking and high BMI increase the severity of symptoms (b=.544, p=0.019, and b=.107, p=0.000, respectively). In contrast, both physical activity and high social support decrease the severity of symptoms (b=-.237, p=0.049 and b=-.239, p=0.001, respectively).

**Table 6:** Significant predictors of the menopausal rating scale and its subscales BMI: Body mass index, CI: Confidence interval, LL: Lower limit, UL: Upper limit

	B	T	p-value	95% CI	
				LL	UL
Model 1: Total menopausal rating score					
BMI	.352	5.919	0.000	.235	.469
Total social support score	-.600	2.704	0.007	-.745	-.164
Regularity of menses	2.526	2.827	0.005	1.76	4. 28
Smoking	3.943	2.680	0.008	1.04	6.83
Physical activity	-1.065	1.984	0.048	-2.12	-1.4
Model 2: Somatic subscale					
Age	.070	2.161	0.031	.006	.133
Smoking	.579	2.023	0.044	.016	.942
BMI	.096	3.912	0.000	.048	.145
Regularity of cycle	.608	2.798	0.005	.181	.956
Model 3: Psychological subscale					
Smoking	.839	2.495	0.013	.178	.995
Physical activity	-.637	2.488	0.013	-.840	-.134
Total social support score	-.419	3.953	0.000	-.628	-.211
Model 4: Urogenital subscale					
BMI	.107	5.342	0.000	.068	.146
Smoking	.544	2.349	0.019	.088	.990
Physical activity	-.237	1.967	0.049	-.588	-.114
Total social support score	-.239	-3.241	0.001	-.383	-.094

## Discussion

In the present study, the severity of menopausal complaints was assessed among Saudi women aged 45 and older using the MRS, and it had a good reliability (= 0.880) in our sample. According to our data, the most common symptoms were physical and mental exhaustion (80.3%), joint and muscular stiffness (79.2%), and irritability (75.9%). This result is in line with the findings of other studies [19-23]. This was especially true in the Gulf region [24]. The high percentage of physical and mental exhaustion in menopausal symptoms could be explained by the fluctuation of hormones during this critical period of women’s life which led to mood changes and even depressive moods [3]. The high prevalence of joint and muscular discomfort among our sample could be attributed to several factors such as the high percentage of obesity in our sample and low physical activity [25]. In addition, vitamin D insufficiency is common among Saudi women and contributes to joint and muscle pain [26].

The results of our study was a little different from studies conducted in both Sri Lanka and Malaysia in which the severity of symptoms was lower and the most common symptoms were hot flashes and sweating which could be explained by the racial and ethnic difference in addition to the fact that the women here were leaner [21,22]. The mean body mass index (BMI) among our participants was 30.1 which was higher than reported in other studies published elsewhere [27-29]. This could be explained by the low percentage of physical activity among our target population. However, this excess weight raises a concern about a public health problem in that age group in which there will be more liability for other comorbidities such as heart diseases.

According to our results on the mean menopause rating scale, the symptoms were moderate in severity ( 13.7±8.3) which was matched with the findings of other studies [20,30] and also consistent with another study done in Saudi Arabia where their score of 15.6 is located in the same range of moderate severity [31]. The results of the current study showed that the socio-demographic variables such as marital status, education and occupation had a non-significant effect on the severity of menopausal symptoms as documented in the results of linear regression. This was similar to the results of other literature [32,33]. However, only older age was significantly associated with the severity of somatic symptoms in our results and was in accordance with a study conducted in Al Hassa governorate in Saudi Arabia [34].

There is evidence from previous studies that higher the BMI higher the reported menopausal symptoms. Our results showed that the severity of the somatic and urogenital symptoms increased significantly with increased BMI and this association persists after controlling for confounders in multiple linear regression which is in line with the results of several studies [35-39]. Our study showed also that obesity was significantly associated with an increase in total MRS score as documented in the Brazilian study [40]. This association is attributed to hemodynamic and anatomic cardiac changes, hormonal and metabolic changes, inflammation, and comorbidities resulting from excess body fat. In many previous studies, smoking has been linked to the severity of menopausal symptoms especially vasomotor symptoms and even the occurrence of early menopause due to its anti-estrogenic effect [41,42]. The findings of our study add to the scientific proof that smoking is linked with an increase in severity of all symptoms of menopause as somatic, psychological and urogenital.

The results of linear regression demonstrated that after controlling for confounders, physical activity was associated with the reduction in the severity of the total MRS score and the psychological and urogenital sub-scales. This finding was matched with findings from additional investigations [43,44]. There is evidence that PA improves self-esteem and relieves stress. Exercise is an efficient treatment for moderate depression and anxiety and this explains the alleviation of the psychological symptoms during menopause aside from its indirect effect through obesity reduction [25,45].

According to our knowledge, this was the first study to assess the association between the perceived social support and severity of menopausal symptoms among Saudi women. Regression analysis showed that social support was an important determinant of the severity of menopausal symptoms, especially both psychological and urogenital menopausal symptoms. It acts as a buffering mechanism. Elsewhere in the literature there are reports that various kinds of support (familial, emotional, etc.) are associated with fewer menopause symptoms which support our finding [46,47].

The average MSPSS score in this study was 4.3, which is considered medium perceived social support. This finding is consistent with other publications’ findings [48,49]. The women received high levels of social support from their families, but only moderate levels of support from friends and significant other subscales. Other studies, however, found that females had a higher level of perceived social support [48-50]. It is not a stretch to say that the majority of Saudi females reported high social support from family, which could be explained by the fact that in Saudi cultures, women live with their husbands and children, have communication and support within the family, share symptoms with family members, and share information about symptoms management. However, the role of receiving social support from friends and significant other persons was moderate. There is a need for more social activity for Saudi women as well as the necessity for help from the social council through health care workers such as nursing staff to alleviate menopausal symptoms.

Limitation and strength

The nature of this cross-section study may introduce a recall bias. However, the use of a valid reliable questionnaire alleviates this bias. Also, we cannot establish a causal relationship between the detected significant effect of lifestyle factors and the severity of menopausal symptoms by cross-section design. The sample was selected randomly using a random sampling technique and an adequate sample size was fulfilled to ensure a good representative sample.

## Conclusions

Middle-aged Saudi women reported mild to moderate menopausal symptoms. Our findings add to a growing body of evidence linking smoking, obesity, and a lack of physical activity to a worsening of menopausal symptoms. Social support was also discovered to have a substantial impact on lowering menopausal symptoms or functioning as a buffer mechanism to ease psychological distress at this key phase in a woman's life. This has an implication for clinical practices and will be useful in figuring out how to manage and intervene with menopausal symptoms. Premenopausal counseling for women includes advice from health care professionals on changing women's lifestyles including stopping smoking early, weight reduction, physical activity and engagement in social activities. Future studies should look into whether social interventions have the desired effect on menopause symptoms and if different personality types experience menopause differently.

## Appendices

Structured anonymous pre-tested questionnaire for women who met inclusion criteria

Section 1: Demographic data

Section 2: Menopause rating scale

Section 3: Physical activity 

Section 4: Multidimensional scale of perceived social support

Section 1

Demographic Data, Reproductive History, Lifestyle Factors, and Chronic Disease and Anthropometric Measurements

1. Demographic data

·   Age 

·   Marital status:

     Single

     Married

     Divorced

     Widowed

·   Education level:

    Uneducated/primary

    Intermediate

    Secondary

    University

·  Occupation:

    Working 

    Not working

2. Reproductive history

·  Fertility

   Given birth 

   Not given birth

·  Describe menstrual regularity in the past 12 months

   Regular 

   Irregular

3. Lifestyle factors

·   Smoking

    Non-smoker

    Current smoker

    Ex-smoker

4. Chronic disease and anthropometric measurements

·    Weight

·    Height

·    Do you have a chronic disease? If yes, what is the disease?

Section 2

Menopause Rating Scale

Please mark the appropriate score for each symptom. For the symptom that does not apply, please mark (none).

                                                                                             None        Mild      Moderate   Severe   Very severe I------------ I-------------I-------------I ------------I 

                                                                                             Score=0               1               2              3               4 

1.     Hot flashes, sweating (episodes of sweating) ………………………….                                  

0               1               2              3               4 

2. Heart discomfort (unusual awareness of heartbeat, heart skipping, heart racing, tightness)                             

0               1               2              3               4 

3. Sleep problems (difficulty in falling asleep, difficulty in sleeping through the night, waking up early)             

0               1               2              3               4 

4. Depressive mood (feeling down, sad, on theverge of tears, lack of drive, mood swings)                                   

0               1               2              3               4 

5. Irritability (feeling nervous, inner tension, feeling aggressive)                                                                         

0               1               2              3               4 

6. Anxiety (inner restlessness, feeling panicky)                           

0               1               2              3               4 

7. Physical and mental exhaustion (general                                                                                                                                 decrease  in performance, impaired memory, decrease                                                                                                           in concentration, forgetfulness)                                                      

0               1               2              3               4 

8. Sexual problems (change in sexual desire, in                                                                                                                                 sexual activity and satisfaction)                                                       

0               1               2              3               4 

9. Bladder problems (difficulty in urinating,                                                                                                                                        increased need to urinate, bladder incontinence)                        

0               1               2              3               4 

10. Dryness of vagina (sensation of dryness or burning                                                                                                                      in the vagina, difficulty with sexual intercourse)  

0               1               2              3               4 

11.  Joint and muscular discomfort (pain in the joints,                                                                                                                          rheumatoid complaints)    

0               1               2              3               4 

Section 3

International Physical Activity Questionnaire

We are interested in finding out about the kinds of physical activities that people do as part of their everyday lives. The questions will ask you about the time you spent being physically active in the last 7 days. Please answer each question even if you do not consider yourself to be an active person. Please think about the activities you do at work, as part of your house and yard work, to get from place to place, and in your spare time for recreation, exercise or sport. 

Think about all the vigorous activities that you did in the last 7 days. Vigorous physical activities refer to activities that take hard physical effort and make you breathe much harder than normal. Think only about those physical activities that you did for at least 10 minutes at a time. 

1. During the last 7 days, how many days did you do vigorous physical activities like heavy lifting, digging, aerobics, or fast bicycling? 

_____ days per week.

No vigorous physical activities >>Skip to question 3 

2. How much time did you usually spend doing vigorous physical activities on one of those days? 

_____ hours per day _____ minutes per day 

Don’t know/Not sure 

Think about all the moderate activities that you did in the last 7 days. Moderate activities refer to activities that take moderate physical effort and make you breathe somewhat harder than normal. Think only about those physical activities that you did for at least 10 minutes at a time. 

3. During the last 7 days, how many days did you do moderate physical activities like carrying light loads, bicycling at a regular pace, or doubles tennis? Do not include walking. 

_____ days per week No moderate physical activities>> Skip to question 5 

4. How much time did you usually spend doing moderate physical activities on one of those days? 

_____ hours per day _____ minutes per day

Don’t know/Not sure

Think about the time you spent walking in the last 7 days. This includes at work and at home, walking to travel from place to place, and any other walking that you have done solely for recreation, sport, exercise, or leisure. 

5. During the last 7 days, how many days did you walk for at least 10 minutes at a time? 

_____ days per week

No walking>> Skip to question 7 

6. How much time did you usually spend walking on one of those days

     _____ hours per day _____ minutes per day 

Don’t know/Not sure 

The last question is about the time you spent sitting on weekdays during the last 7 days. Include time spent at work, at home, while doing course work, and during leisure time. This may include time spent sitting at a desk, visiting friends, reading, or sitting or lying down to watch television. 

7.     During the last 7 days, how much time did you spend sitting on a weekday?

 _____ hours per day  _____ minutes per day 

Don’t know/Not sure 

Section 4

Multidimensional Scale of Perceived Social Support

We are interested in how you feel about the following statements.  Read each statement carefully.   Indicate how you feel about each statement.

                                    Circle the “1”  if you Very Strongly Disagree

                                    Circle the “2”  if you Strongly Disagree

                                    Circle the “3”  if you Mildly Disagree

                                    Circle the “4”  if you are Neutral

                                    Circle the “5”  if you Mildly Agree

                                    Circle the “6”  if you Strongly Agree

                                    Circle the “7”  if you Very Strongly Agree

1.     There is a special person who

         is around when I am in need.                                  1         2          3         4       5        6        7

2.     There is a special person with

        whom I can share joys and sorrows.                           1         2          3         4       5        6        7

3.     My family really tries to help me.                               1         2          3         4       5        6        7

4.    I get the emotional help & support

        I need from my family.                                                  1         2          3         4       5        6        7

5.     I have a special person who is

        a real source of comfort to me.                                   1         2          3         4       5        6        7

6.     My friends really try to help me.                                  1         2          3         4       5        6        7

7.     I can count on my friends when

        things go wrong.                                                           1         2          3         4       5        6        7

8.     I can talk about my problems with

        my family.                                                                        1         2          3         4       5        6        7

9.     I have friends with whom I can

        share my joys and sorrows.                                          1         2          3         4       5        6        7

10.   There is a special person in my    

        life who cares about my feelings.                               1         2          3         4       5        6        7

11.   My family is willing to help me

        make decisions.                                                              1         2          3         4       5        6        7

12.   I can talk about my problems with

        my friends.                                                                     1         2          3         4       5        6        7 
